# A mathematical model of aortic aneurysm formation

**DOI:** 10.1371/journal.pone.0170807

**Published:** 2017-02-17

**Authors:** Wenrui Hao, Shihua Gong, Shuonan Wu, Jinchao Xu, Michael R. Go, Avner Friedman, Dai Zhu

**Affiliations:** 1 Department of Mathematics, Pennsylvania State University, State College, PA, United States of America; 2 Beijing International Center for Mathematical Research, Peking University, Beijing, China; 3 Division of Vascular Diseases & Surgery, The Ohio State University Wexner Medical Center, Columbus, OH, United States of America; 4 Department of Mathematics, The Ohio State University, Columbus, OH, United States of America; 5 Yunnan Cocreative Scientific Computing and Datamining Center, Kunming, China; University of Brescia, ITALY

## Abstract

Abdominal aortic aneurysm (AAA) is a localized enlargement of the abdominal aorta, such that the diameter exceeds 3 cm. The natural history of AAA is progressive growth leading to rupture, an event that carries up to 90% risk of mortality. Hence there is a need to predict the growth of the diameter of the aorta based on the diameter of a patient’s aneurysm at initial screening and aided by non-invasive biomarkers. IL-6 is overexpressed in AAA and was suggested as a prognostic marker for the risk in AAA. The present paper develops a mathematical model which relates the growth of the abdominal aorta to the serum concentration of IL-6. Given the initial diameter of the aorta and the serum concentration of IL-6, the model predicts the growth of the diameter at subsequent times. Such a prediction can provide guidance to how closely the patient’s abdominal aorta should be monitored. The mathematical model is represented by a system of partial differential equations taking place in the aortic wall, where the media is assumed to have the constituency of an hyperelastic material.

## Introduction

AAA is an abnormal dilatation most commonly of the infrarenal aorta. One definition of AAA is a diameter greater than 3 cm [1]. The clinical significance of AAA stems from the high mortality associated with rupture. Approximately 60% of patients with ruptured AAA die before reaching the hospital [2] and mortality rates of emergency surgical repair are as high as 35–70% [3]. The risk of rupture increases with AAA diameter. The pathogenesis of AAA is largely unknown and likely multifactorial. Diameter remains the only clinically useful and available marker for risk of rupture. Surgical repair is recommended for aneurysms measuring greater than 5.5 cm [4, 5] and there is insufficient evidence to recommend surgery for all patients with smaller AAA [2]. However, even elective surgical repair of AAA remains a major operation, incurring significant morbidity and mortality particularly in older, sicker patients where there is a higher prevalence of AAA. Furthermore, patients die every year from rupture of aneurysms smaller than 5.5 cm, and up to 60% of AAA larger than 5 cm remain stable [6]. Thus, some patients with smaller AAA are denied lifesaving surgery and others with larger AAA undergo unnecessary major surgery. The reasons why some smaller aneurysms go on to rupture while some larger ones remain stable are not understood. Techniques that provide early identification of small AAA with increased risk for rupture and large AAA with low risk for rupture will improve overall mortality by prompting personalized treatment plans for AAA.

There are a number of mathematical papers that describe the dynamics of the weakening and dilation of the arterial wall and the risk of rupture. The more comprehensive models include the nonlocal, nonlinear elastic nature of the arterial wall in response to wall shear stress [7–10]. However these models do not explain the clinical fact that some abdominal aortae rupture when the diameter of the cross section of the dilated aorta is less than 5.5 cm while others do not rupture even when much larger than 5.5 cm. There is a need to discover non-invasive biomarkers that will provide the following prognosis for a patient undergoing initial screening for AAA:

- Given initial diameter of the cross section of the aorta, say *R*_0_, how will the diameter, say *R*(*t*), evolve with time?
- How will the risk of rupture increase with time?

The present paper develops a mathematical model that addresses the first question, with IL-6 as the serum biomarker. IL-6 has already been suggested as prognostic biomarker for AAA in [11, 12]; additional potential biomarkers are reviewed in [13, 14].

The mathematical model consists of two parts. The first part focuses on the biology. It introduces a network of cells and cytokines which, in disease state, lead to the destruction of smooth muscle cells (SMCs) and elastin depletion in the media, and disruption of the extracellular matrix (ECM) in the adventitia. The biological predictions of this part of the model, in terms of the expression levels of the proteins involved, are in agreement with patients data [15]. The second part of the model develops the mechanics of diameter growth of the artery as a result of deficiency in elastin and disruptions in the ECM. Here, instead of the approach taken in [7–10], we use the fact that the arterial wall behaves like a hyperelastic material [16]. Hence its elastic strength and displacement under stress depend on the smooth muscle cells in the aortic wall. We can therefore relate the wall dilation to several cytokines whose expression is upregulated in disease state. Such cytokines could be used as potential biomarkers for predicting the arterial wall dilation. We shall focus on IL-6, which is upregulated in AAA patients [11, 12].

The biological network introduced in the present paper includes macrophages, T cells, SMCs and fibroblasts, as well as cytokines such as IL-6, IL-10, IL-12, TNF-*α* and IFN-*γ*; MMP and TIMP are also included. The densities of the cells and the concentrations of the proteins satisfy a coupled system of partial differential equations (PDEs) in the arterial wall.

In the early stage of aneurysm, endothelial cells, under hemodynamic shear stress, secrete major attractant protein, MCP-1, and IL-6 [17]. IL-6 is also produced by SMCs (in conjunction with IL-1) [18]. This triggers recruitment of monocytes from the blood into the media layer of the arterial wall [19]. The monocytes mature into macrophages, while, at the same time, additional macrophages from the adventitia are also chemoattracted by MCP-1, IL-6 and IL-8 [20–22]. More precisely, IL6/MCP-1 combined, and MCP-1 and IL-8 separately chemoattract monocytes; for simplicity, we shall represent the chemoattractants by MCP-1 and IL-6. T cells are activated by contact with macrophages in the presence of IL-12, and macrophages are activated by IFN-*γ* produced by the T cells [23]. Macrophages produce TNF-*α* [24], MMP and TIMP [24, 25], and IL-10, IL-12 and IL-6 [26], but MMP is also produced by SMCs [27]. Fibroblasts produce collagen [28], and the collection of MMP, TIMP and collagen weaken the ability of the adventitia layer to withstand stress. Macrophages are known to cause apoptosis in SMCs [19], and this leads to reduction in elastin [29], thus weakening the elastic strength of the media; The apoptotic SMCs are known to produce MCP-1 [19].

**Fig 1:**
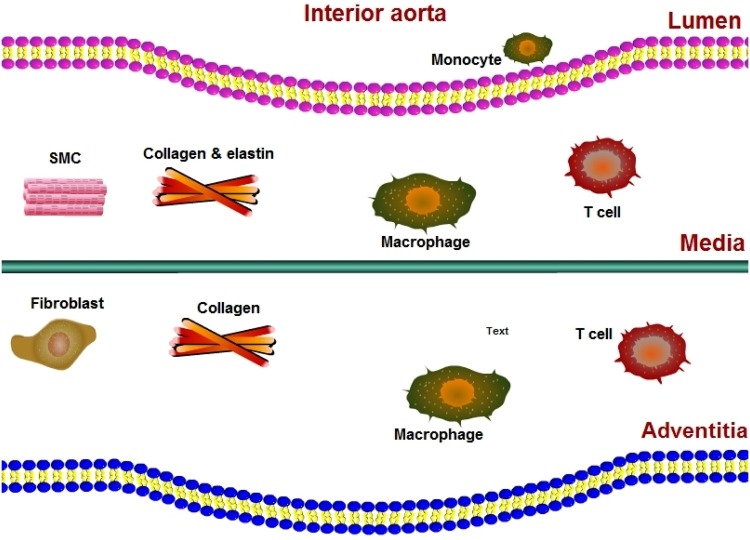
A schematic of the aortic wall including the aortic lumen, the intima, media, and adventitia.

Fig 1 shows SMCs residing in the media, fibroblasts residing in the adventitia, while macrophages and T cells are present in both layers of the aortic wall.

**Fig 2:**
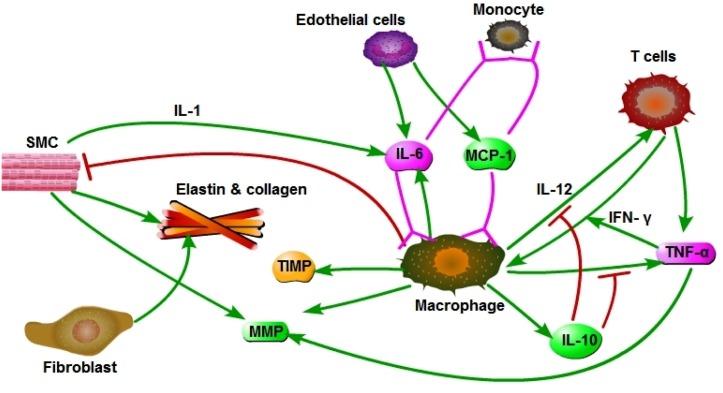
Interaction network among cells and cytokines in their respective layers. SMCs remain in the media, and fibroblasts remain in the adventitia.

**Fig 3:**
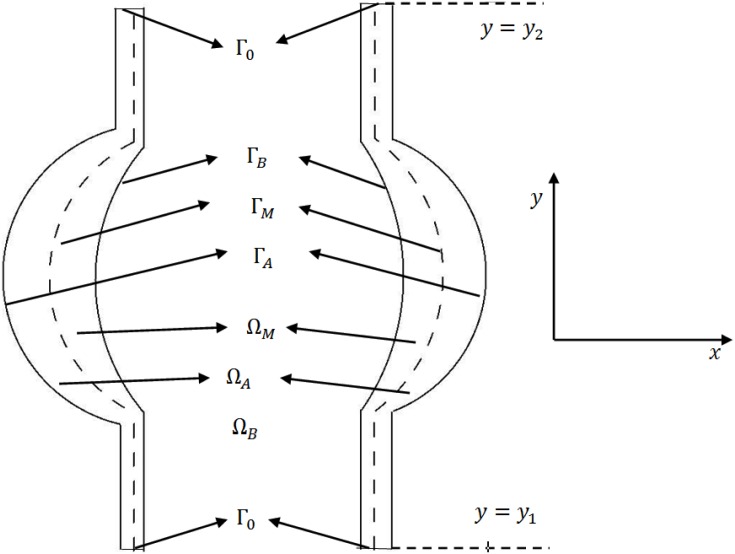
Schematic representation of a 2D section of the computational domain. Ω_*B*_ is blood vessel; Ω_*M*_ represents the media and Ω_*A*_ the adventitia; Γ_*A*_, Γ_*B*_ and Γ_*M*_ are three free boundaries. Γ_*B*_ is the inner surface of the artery, Γ_*A*_ is the outer surface, and Γ_*M*_ is the surface between the media and adventitia.

Fig 2 is a schematics of the network within the arterial wall during aneurysm; all the cytokines and the ECM (elastin and collagen) are present in both the media and the adventitia. A schematics of the balloon-like bulge geometry in the aorta is shown in Fig 3.

## 1 Mathematical model of the biology

In this section we develop a mathematical model of aneurysm based on the diagram shown in Fig 2. The model, represented by a system of PDEs, includes the variables listed in Table 1. We assume that all cells are moving with a common velocity **v**; the velocity is the result of movement of macrophages, T cells and SMCs into the media and adventitia. We also assume that all species are diffusing with appropriate diffusion coefficients. The equation for each species of cells, *X*, has a form

$$\begin{array}{c} \frac{dX}{dt}+\nabla \cdot \left(vX\right)-D_{X}\Delta X=F_{X}, \end{array}$$

where the expression on the left-hand side includes advection and diffusion, and *F*_*X*_ accounts for various biochemical reactions, and chemotaxis. The equations for the chemical species are the same as for cells, but without the advection term, which is relatively very small compared to their large diffusion coefficients. Fig 3 shows a 2D projection of a blood vessel Ω_*B*_ with an aortic aneurysm Ω_*M*_ ∪ Ω_*A*_.

**Table 1:** The variables of the model; concentration and densities are in units of *g*/*cm*^3^.

*S*:	density of SMCs	*T*:	density of T cells (Th1)
*M*:	density of macrophages	*f*:	density of fibroblasts
*ρ*:	density of ECM	*P*:	concentration of MCP-1
*T*_*α*_:	concentration of TNF-*α*	*I*_*γ*_:	concentration of INF-*γ*
*I*_6_:	concentration of IL-6	*I*_10_:	concentration of IL-10
*I*_12_:	concentration of IL-12	*Q*:	concentration of MMPs
*Q*_*r*_:	concentration of TIMP	*p*:	pressure in media (in *g*/*cm*/*day*^2^)
**u**:	displacement (in *cm*)	**v**:	cell velocity in media and adventitia (in *cm*/*day*)

### Equation for MCP-1 (*P*)

The equation for MCP-1 is given in the whole domain (media Ω_*M*_, and adventitia Ω_*A*_) by

$$\begin{array}{ccc} \frac{\partial P}{\partial t}-D_{P}\Delta P & = & \underset{production}{\underset{︸}{\lambda_{PS}\frac{M}{M+K_{M}}S}}\underset{degradation}{\underset{︸}{-d_{PM}\frac{P}{P+K_{P}}M-d_{P}P}}, \end{array}$$ (1)

The first term on the right-hand side is the production of *P* by apoptotic SMCs, which is induced by macrophages [19]. The second term represents lost of MCP-1 as it binds to, and internalized by, macrophages which are chemoattracted to MCP-1; the internalization of MCP-1 may be limited due to the limited rate of receptor recycling. The last term in Eq (1) accounts for degradation of MCP-1.

### Equation for macrophages (*M*)

According to [26] the two phenotype of macrophages, M1 and M2, are both present in aneurysm; M1 secretes inflammatory cytokines such as IL-12 and TNF-*α*, while M2 secretes immunoregulatory cytokines such as IL-10. For simplicity, we do not differentiate between the two phenotypes, but take into account that the productions of IL-12 and TNF-*α* are resisted by IL-10 [30].

The evolution of the macrophage density in Ω_*M*_ ∪ Ω_*A*_ is modeled by

$$\begin{array}{ccc} \frac{\partial M}{\partial t}+\nabla \cdot \left(vM\right)-D_{M}\Delta M & = & \underset{chemotaxis}{\underset{︸}{-\nabla \cdot \left(M\chi_{C}\nabla P\right)-\nabla \cdot \left(M\chi_{C}\nabla I_{6}\right)}}\\ & & +\underset{activation}{\underset{︸}{\lambda_{MI_{\gamma }}M\frac{I_{\gamma }}{I_{\gamma }+K_{I_{\gamma }}}(1+\lambda_{MT_{\alpha }}\frac{T_{\alpha }}{T_{\alpha }+K_{T_{\alpha }}})}}\underset{death}{\underset{︸}{-d_{M}M}}.\;\; \end{array}$$ (2)

The first two terms on right-hand side account for the recruitment of macrophages by MCP-1 [19] and IL-6 [20–22]. The third term accounts for the activation of macrophages by IFN-*γ* [23], which is enhanced by TNF-*α* [31].

### Equation for T cells (*T*)

The density of T cells in Ω_*M*_ ∪ Ω_*A*_ satisfies the equation

$$\begin{array}{ccc} \frac{\partial T}{\partial t}+\nabla \cdot \left(vT\right)-D_{T}\Delta T & = & \underset{activtion}{\underset{︸}{\lambda_{T}\frac{I_{12}}{1+I_{10}/K_{I_{10}}}M}}\underset{death}{\underset{︸}{-d_{T}T}}. \end{array}$$ (3)

We assume that T cells are activated by contact with macrophages in IL-12 environment, while IL-12 is inhibited by IL-10 [30].

### Equation for SMCs (*S*)

The equation of the SMCs density in Ω_*M*_ is given by

$$\begin{array}{ccc} \frac{\partial S}{\partial t}+\nabla \cdot \left(vS\right)-D_{S}\Delta S & = & \lambda_{S}\underset{apoptosis}{\underset{︸}{-d_{SM}\frac{M}{M+K_{M}}S}}-d_{S}S.\;\; \end{array}$$ (4)

The second term of the right-hand side accounts for apoptosis of SMCs, caused by macrophages through a FasL/Fas-Caspase8-RIP1 mediated mechanism [19].

### Equations for cytokines (in Ω_*M*_ ∪ Ω_*A*_)

IL-6 is produced by macrophages [26], and by SMCs (in conjunction with IL-1) [18]; hence

$$\begin{array}{ccc} \frac{\partial I_{6}}{\partial t}-D_{I_{6}}\Delta I_{6} & = & \underset{production}{\underset{︸}{\lambda_{I_{6}M}M+\lambda_{I_{6}S}S}}\underset{degradation}{\underset{︸}{-d_{I_{6}}I_{6}}}. \end{array}$$ (5)

IL-10 is produced by macrophages [26], so that

$$\begin{array}{ccc} \frac{\partial I_{10}}{\partial t}-D_{I_{10}}\nabla^{2}I_{10} & = & \underset{production}{\underset{︸}{\lambda_{I_{10}M}M}}\underset{degradation}{\underset{︸}{-d_{I_{10}}I_{10}}}. \end{array}$$ (6)

IL-12 is produced by macrophages [26], a process inhibited by IL-10. The equation for *I*_12_ is given by

$$\begin{array}{ccc} \frac{\partial I_{12}}{\partial t}-D_{I_{12}}\nabla^{2}I_{12} & = & \underset{\text{production}\;\text{by}\;\text{macrophage}}{\underset{︸}{\lambda_{I_{12}M}\frac{M}{1+I_{10}/K_{I_{10}}}}}\underset{degradation}{\underset{︸}{-d_{I_{12}}I_{12}}}. \end{array}$$ (7)

Similarly, TNF-*α* is produced by macrophages, a process inhibited by IL-10 [32], so that

$$\begin{array}{ccc} \frac{\partial T_{\alpha }}{\partial t}-D_{T_{\beta }}\Delta T_{\alpha } & = & \underset{production}{\underset{︸}{\lambda_{T_{\alpha }M}\frac{M}{1+I_{10}/K_{I_{10}}}}}\underset{degradation}{\underset{︸}{-d_{T_{\alpha }}T_{\alpha }}}. \end{array}$$ (8)

IFN-*γ* is produced by T cells [33], hence

$$\begin{array}{ccc} \frac{\partial I_{\gamma }}{\partial t}-D_{I_{\gamma }}\Delta I_{\gamma } & = & \underset{production}{\underset{︸}{\lambda_{I_{\gamma }}T}}\underset{degradation}{\underset{︸}{-d_{I_{\gamma }}I_{\gamma }}}. \end{array}$$ (9)

MMP is produced by macrophages [25] and by SMCs [34], a process enhanced by TNF-*α* [24], and MMP is lost by binding with TIMP [25]. Accordingly, *Q* satisfies the following equation:

$$\begin{array}{ccc} \frac{\partial Q}{\partial t}-D_{Q}\Delta Q & = & \underset{production}{\underset{︸}{\left(\lambda_{QM}M+\lambda_{QS}S\right)(1+\lambda_{QT_{\alpha }}\frac{T_{\alpha }}{T_{\alpha }+K_{T_{\alpha }}})}}\underset{depletion}{\underset{︸}{-d_{QQ_{r}}Q_{r}Q}}\underset{degradation}{\underset{︸}{-d_{Q}Q}},\; \end{array}$$ (10)

TIMP is produced by macrophages [25], and is lost by binding to MMP, so that

$$\begin{array}{ccc} \frac{\partial Q_{r}}{\partial t}-D_{Q_{r}}\Delta Q_{r} & = & \underset{production}{\underset{︸}{\lambda_{Q_{r}M}M}}\underset{depletion}{\underset{︸}{-d_{Q_{r}Q}QQ_{r}}}\underset{degradation}{\underset{︸}{-d_{Q_{r}}Q_{r}}}. \end{array}$$ (11)

### Equation for ECM (*ρ*)

SMCs produce elastin [29] and fibroblasts produce collagen [28]. We assume that the density of fibroblasts is approximately constant, and that the ECM concentration is proportional to the combined concentrations of collagen and elastin, which are produced by fibroblasts and SMCs, respectively. Hence,

$$\begin{array}{c} \frac{\partial \rho }{\partial t}+\nabla \cdot \left(v\rho \right)-D_{\rho }\Delta \rho \;=\;\underset{production}{\underset{︸}{[\lambda_{\rho f}\chi_{A}+\lambda_{\rho S}S]{\left(1-\frac{\rho }{\rho_{0}}\right)}^{+}}}\underset{degradation}{\underset{︸}{-d_{\rho }\rho -d_{\rho Q}Q\rho }}, \end{array}$$ (12)

where the term *d*_*ρQ*_ *Qρ* represents ECM degradation by MMP. Here we used the notations: *X*^+^ = *X* if *X* > 0, *X*^+^ = 0 if *X* ≤ 0, and *χ*_*A*_ = 1 in Ω_*A*_, *χ*_*A*_ = 0 in Ω_*M*_.

### Boundary conditions

Fig 3 shows a 2-D projection of the media and adventitia layers of the arterial wall. We shall take as our computational domain the right region lying between *y* = *y*_1_ and *y* = *y*_2_, and impose periodic boundary condition at *y* = *y*_1_ and *y* = *y*_2_.

T cells, which are primarily CD4+ Th1 cells [35], migrate to the adventitia and intima [36, 37], and so do macrophages [22, 37].

The concentration *ρ* is continuous across the media/adventitia membrane, while *S* satisfies no-flux boundary condition, $\frac{\partial S}{\partial n}=0$, at this membrane. All the other variables satisfy the following flux conditions at the interface Γ_*M*_:

$$\begin{array}{c} \frac{\partial X_{M}}{\partial n}+\gamma \left(X_{M}-X_{A}\right)=0,\;\text{and}\;\frac{\partial X_{A}}{\partial n}+\gamma \left(X_{A}-X_{M}\right)=0, \end{array}$$

where *X*_*M*_ and *X*_*A*_ are the concentrations of cytokines or cell densities in media and adventitia, respectively. We take *γ* = 50 for cells, and *γ* = 500 for the cytokines [38].

On Γ_*B*_ and Γ_*A*_, we have

$$\begin{array}{c} \frac{\partial M}{\partial n}+\alpha_{M}\left(P\right)\left(M-M_{0}\right)=0,\;\frac{\partial T}{\partial n}+\alpha_{T}\left(T-T_{0}\right)=0, \end{array}$$

where $\alpha_{M}\left(P\right)={\tilde{\alpha }}_{M}\frac{P}{P+K_{P}}$.

Both MCP-1 and IL-6 are produced by endothelial cells [17], which lie near the inner boundary of the media, Γ_*B*_. Hence

$$\begin{array}{c} \frac{\partial P}{\partial n}+\alpha_{P}\left(P-P_{0}\right)=0,\;\frac{\partial I_{6}}{\partial n}+\alpha_{I_{6}}\left(I_{6}-{I_{6}}_{0}\right)=0\;\text{on}\;\Gamma_{B}, \end{array}$$ (13)

where *P*_0_ and *I*_60_ are the concentrations of MCP-1 and *I*_6_ in the blood. We assume no-flux boundary conditions on Γ_*B*_ for all remaining variables. We also assume no-flux conditions on Γ_*A*_ for all variables (except *M* and *T*, as stated above)

### Initial conditions

We take initially $I_{6}=\frac{I_{60}}{2}$, where *I*_60_ = 6 × 10^−9^ *gcm*^−3^ in the source of influx of *I*_6_ from the endothelial cells [39], and as in [40],

$$\begin{array}{c} Q=3\times 10^{-8},\;Q_{r}=10^{-8},\;\rho =3.43\times 10^{-4}\;\text{and}\;S=S_{0},\;\text{where}\;S_{0}=6\times 10^{-3}\;gcm^{-3}. \end{array}$$

All other cell densities and cytokine concentrations are taken to be zero initially. We prescribe the initial geometry of aneurysm by

$$\begin{array}{c} \Gamma_{A}:\;x=0.7\cos{\left(\pi y\right)}^{2}+0.3,\;\Gamma_{M}:\;x=0.55\cos{\left(\pi y\right)}^{2}+0.2,\\ \Gamma_{B}:\;x=0.5\cos{\left(\pi y\right)}^{2}\;\text{for}\;y_{1}\leq y\leq y_{2}. \end{array}$$

## Mechanical hyperelasticity model

We set Ω = Ω_*M*_ ∪ Γ_*M*_ ∪ Ω_*A*_ and refer to this region, briefly, as the aortic wall. Aortic wall is assumed to have nonlinear, hyperelastic material properties [27, 41, 42]. Specifically, it is an ‘almost’ incompressible, homogeneous, and isotropic material with energy density function *W* of the form

$$\begin{array}{c} W=\beta_{1}\left(I_{B}-3\right)+\beta_{2}{\left(I_{B}-3\right)}^{2}, \end{array}$$ (14)

where *β*_1_ and *β*_2_ are forces that represent elastic coefficients; **I**_**B**_ is the first invariant of the Left Cauchy-Green tensor **B** (namely **I**_**B**_ = *tr*(**B**)), **B** = **F** **F**^*T*^, while **F** is the deformation gradient tensor. In terms of the displacement vector **u** = (*u*_*i*_), we can write $B={\left[{\left(I-\nabla u\right)}^{T}\left(I-\nabla u\right)\right]}^{-1}={\left(\delta_{ij}-\frac{\partial u_{j}}{\partial x_{i}}-\frac{\partial u_{i}}{\partial x_{j}}+\frac{\partial u_{k}}{\partial x_{i}}\frac{\partial u_{k}}{\partial x_{j}}\right)}^{-1}≐{\left(a_{ij}\right)}^{-1}$, where **I** is the identity tensor.

The Cauchy stress tensor is given by

$$\begin{array}{c} \sigma =-pI+2\frac{\partial W}{\partial I_{B}}B, \end{array}$$ (15)

where *p* is the hydrostatic pressure. The momentum equation is then

$$\begin{array}{ccc} dD_{t}v-\nabla \cdot \sigma & =f & \;\text{in}\;\Omega , \end{array}$$ (16)

where **f** is the body force and *d* is the tissue density. In the sequel we assume no body force and neglect tissue inertia, so that

$$\begin{array}{ccc} \nabla \cdot \sigma & =0, & \;\text{in}\;\Omega . \end{array}$$ (17)

The coefficients *β*_1_ and *β*_2_ are functions of the SMCs which we take to represent the elasticity coefficients. We shall use the following specific relations in Eq (14):

$$\begin{array}{c} \beta_{1}=\beta_{10}+k_{1}(\frac{S}{S_{0}}-1),\;\beta_{2}=\beta_{20}+k_{2}(\frac{S}{S_{0}}-1). \end{array}$$ (18)

The pressure, *p*, is a function of ECM, which we take to have the form

$$\begin{array}{c} p\left(\rho \right)=p^{*}-\beta_{p}(1-\frac{\rho }{\rho^{*}}). \end{array}$$ (19)

for some parameters *β*_*p*_, p*.

For boundary conditions, we take

$$\begin{array}{c} \sigma n=\sigma_{B}n-\gamma \kappa n\;\text{on}\;\Gamma_{B},\\ {\left(\sigma n)\right|}_{\Omega_{M}}={\left(\sigma n)\right|}_{\Omega_{A}}+\gamma \kappa n\;\text{on}\;\Gamma_{M}, \end{array}$$ (20)

$$\begin{array}{c} \sigma n=0\;\text{on}\;\Gamma_{A}, \end{array}$$ (21)

where *σ*_*B*_ is the stress tensor from the blood and *κ* is the mean curvature.

## Results

**Fig 4:**
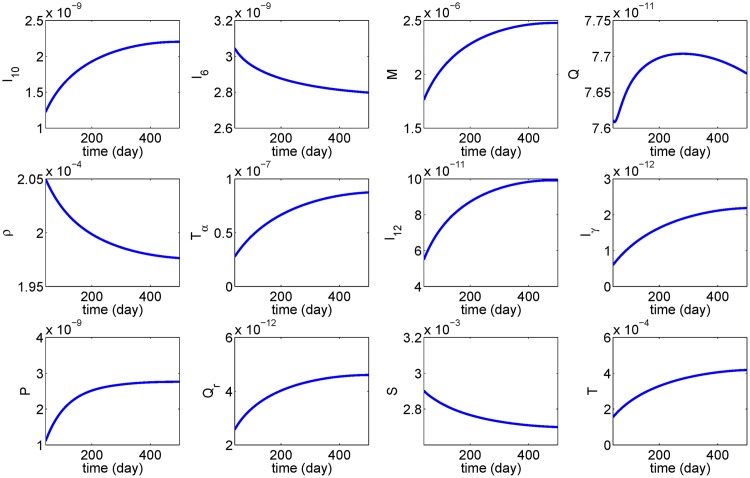
Simulation results over 500 days. The parameter used are as in Tables 2 and 3 with *I*_60_ = 6 × 10^−9^ g/ml.

**Fig 5:**
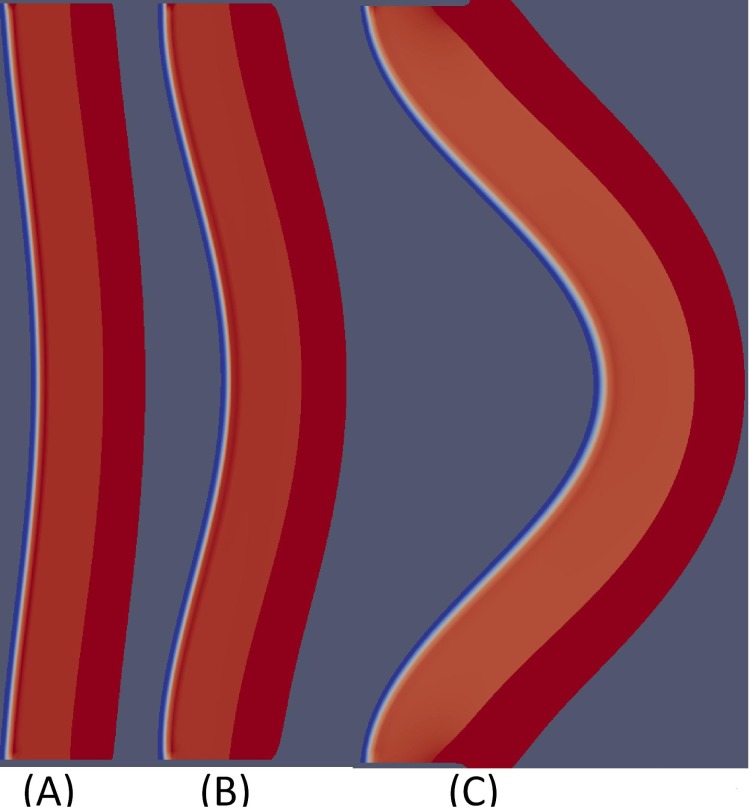
Aneurysm deformation at day 500 with initial deformation shown in (A). The parameters used are as in Tables 2 and 3 with (B) *I*_60_ = 6 × 10^−9^ g/ml, and (C) *I*_60_ = 6 × 10^−8^ g/ml. The diameter grows from 2 cm to 2.5 cm in case (B), and to 4.5 cm in case (C).

Fig 4 shows the profile of average of cell densities and cytokines concentrations in the first 500 days, and Fig 5 shows how the aneurysm deforms at day 500.

From Fig 4, we see that, as AAA progresses, the average densities of SMCs and ECM decrease. The concentration of the inflammatory cytokines TNF-*α*, IFN-*γ* and MCP-1 are increasing. On the other hand, the average concentration of MMP is increasing only for the first 200 days, and it then decreases as the concentration of TIMP keeps growing. The average concentration of IL-6 in the tissue decreases and nearly stabilizes after 500 days. Fig 5 shows growth of the aorta bulge after 500 days: from diameter of 2 cm to diameter of nearly 2.5 cm when *I*_60_ = 6 × 10^−9^ g/ml and nearly 4.5 cm when *I*_60_ = 6 × 10^−8^ g/ml.

In Figs 4 and 5, the initial values of the pro-inflammatory cytokines were taken to be below their steady state. On the other hand the SMCs and ECM densities were taken to be above their steady state. Other choices of the initial conditions yield similar profiles (not shown here).

**Fig 6:**
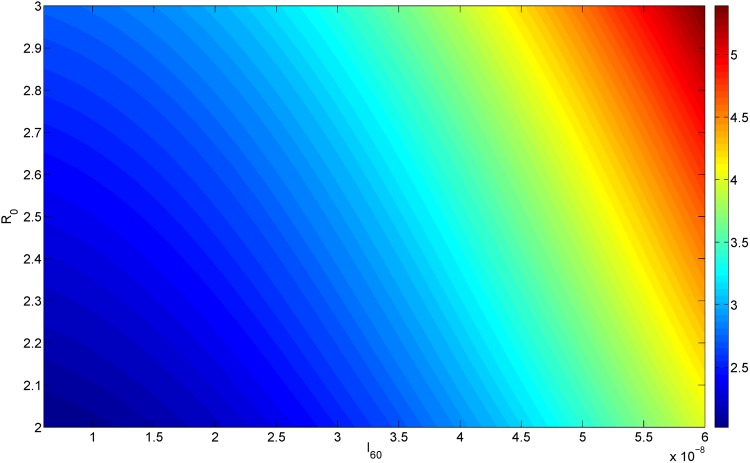
AAA growth. The x-axis scales *I*_60_ from 6 × 10^−9^ g/ml to 6 × 10^−8^ g/ml; the y-axis scales *R*_0_ from 2 cm to 3 cm; The color represents the diameter of aortic bulge at 300 day.

**Fig 7:**
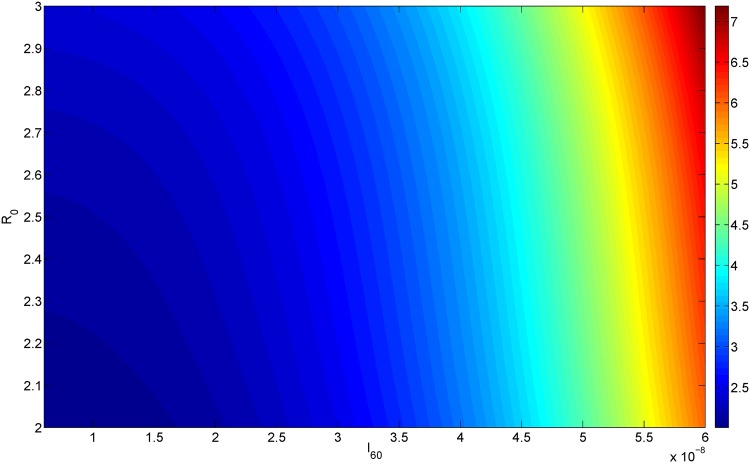
AAA growth. The x-axis scales *I*_60_ from 6 × 10^−9^ g/ml to 6 × 10^−8^ g/ml; the y-axis scales *R*_0_ from 2 cm to 3 cm; The color represents the diameter of aortic bulge at 500 day.

If at the initial screening for AAA we measure both the aorta diameter, *R*_0_, and the concentration of IL-6 in the blood, *I*_60_, then by simulating the model with these values of *R*(0) = *R*_0_ and *I*_60_ (in Eq (13)), we can predict the AAA diameter at any future time *T*. Figs 6 and 7 simulate the cases *T* = 300 and *T* = 500 days. Thus, for every pair of *I*_60_ (on the horizontal axis) and *R*_0_ (on the vertical axis) the color on the column shows the diameter corresponding to the point (*I*_60_, *R*_0_) at day *T* = 300 (upper) and *T* = 500 (lower).

## Conclusions

Abdominal aortic aneurysm is a localized enlargement of the abdominal aorta, which may lead to rupture of the aorta. The disease is asymptomatic until rupture, which is nearly always fatal. The risk of rupture associated with the increased diameter of the aorta varies among people. Hence it is difficult to determine, on the basis of just the current diameter (*R*_0_) of a patient’s aortic wall, how closely this patient’s abdominal aorta should be monitored. In order to make such a determination, we need address the following questions:

- how fast the diameter will grow over time?
- how does the risk of rupture depend on the growing diameter?

Several non-invasive prognostic biomarkers for AAA have been suggested [13, 14]; specifically, IL-6 [11, 12].

The present paper addresses the first question with a mathematical model: Given *R*_0_ and the serum concentration of IL-6, the model predicts the dynamical growth of the diameter, *R*(*t*), for any future time *t*. The mathematical model includes the basic biology underlying AAA formation as a disease that degrades the elastic strength of the aortic wall. Simulations of the model can show how the initial diameter *R*_0_ and the serum concentration of IL-6 determine the diameter *R* after a given period of time T; This is done in Fig 6 in the cases of *T* = 300 days and *T* = 500 days. Thus, the level of IL-6 concentration in the blood can be used to suggest how soon to schedule the next screening of the patient’s abdominal aorta.

The model is represented by a system of partial differential equations, based on some simplified assumptions. In particular, the model assumes that the disease is associated with initial inflammation, and that the aortic wall is a ‘pure’ hyperelastic material. Furthermore, some of the parameters were estimated crudely, for the lack of available data. The authors plan to follow up this study with patients data, which are unavailable at this time. The patients will be followed every 6 months with CT scan and measurement of their aneurysm, and of course any patient who comes for surgery or rupture will be recorded. When patients data become available, the model could then be refined, in particular by adjusting some parameters for specific groups of patients. The model could then be used as a predictive tool for monitoring the enlargement of the abdominal aorta.

## Parameter estimates

All the parameter values are listed in Tables 2 and 3. Most parameters are taken from the literature while some are estimated below. A few remaining parameters are fitted.

**Table 2:** Parameters’ description and value.

Parameter	Description	Value
*D*_*M*_	dispersion coefficient of macrophages	8.64 × 10^−7^ *cm*^2^ day^−1^ [40, 46, 47]
*D*_*T*_	diffusion coefficient of T-cell	8.64 × 10^−7^ *cm*^2^ day^−1^ [40, 46, 47]
*D*_*I*_*γ*__	diffusion coefficient of IFN-*γ*	1.08 × 10^−2^ *cm*^2^ day^−1^ [40, 46, 47]
*D*_*T*_*α*__	diffusion coefficient for TNF-*α*	1.29 × 10^−2^ *cm*^2^ day^−1^ [47]
*D*_*S*_	diffusion coefficient of SMCs	8.64 × 10^−7^ *cm*^2^ day^−1^ [40, 46]
*D*_*P*_	diffusion coefficient of MCP-1	1.728 × 10^−1^ *cm*^2^ day^−1^ [40, 46]
*D*_*Q*_	diffusion coefficient of MMP	4.32 × 10^−2^ *cm*^2^ day^−1^ [40, 46]
*D*_*Q*_*r*__	diffusion coefficient for TIMP	4.32 × 10^−2^ *cm*^2^ day^−1^ [40, 46]
*D*_*I*_6__	diffusion coefficient of IL-10	1.08 × 10^−2^ *cm*^2^ day^−1^ [40, 46, 47]
*D*_*I*_10__	diffusion coefficient of IL-10	1.08 × 10^−2^ *cm*^2^ day^−1^ [40, 46, 47]
*D*_*I*_12__	diffusion coefficient of IL-12	1.08 × 10^−2^ *cm*^2^ day^−1^ [40, 46, 47]
*λ*_*T*_*α*_*M*_	activation rate of TNF-*α* due to macrophage	2.86 × 10^−3^ day^−1^ [47]
*λ*_*MI*_*γ*__	activation rate of macrophages by IFN-*γ*	0.005 day^−1^ [40, 46]
*λ*_*T*_	activation rate of Th1 cells by IL-12	6 × 10^-4^ day^−1^ [47]
*λ*_*I*_6_*M*_	production rate of IL-6 by macrophages	1.73 × 10^−6^ day^−1^ [16] & estimated
*λ*_*I*_6_*S*_	production rate of IL-6 by SMCs	1.73 × 10^−5^ day^−1^ [18] & estimated
*λ*_*I*_*r*_*T*_	production rate of IFN-*γ* by T cells	2.34 × 10^−6^ day^−1^ [47]
*λ*_*I*_12_*M*_	production rate of IL-12 by macrophages	3.78 × 10^−3^ day^−1^ [47]
*λ*_*I*_10_*M*_	production rate of IL-10 by macrophages	2 × 10^−3^ day^−1^ [47]
*λ*_*QM*_	production rate of MMP by macrophages	3 × 10^−4^ day^−1^ [48]
*λ*_*QS*_	production rate of MMP by SMCs	2.16 × 10^−5^ day^−1^ [40, 46]
*λ*_*QT*_*α*__	production rate of MMP by TNF-*α*	2 fitted
*λ*_*Q*_*r*_*M*_	production rate of TIMP by macrophages	6 × 10^−5^ day^−1^ [40, 46]
*λ*_*PS*_	activation rate of MCP-1 due to SMCs	3 × 10^−3^ day^−1^ fitted
*λ*_*ρf*_	activation rate of ECM due to fibroblasts	3 × 10^−4^ day^−1^ [40]
*λ*_*ρS*_	activation rate of ECM due to SMCs	1 × 10^−1^ day^−1^ fitted
*d*_*M*_	death rate of macrophages	0.015 day^−1^ [40, 46, 47]
*d*_*ρ*_	degradation rate of ECM	0.37 day^−1^ [40, 46, 47]
*d*_*P*_	degradation rate of MCP-1	1.73 day^−1^ [40, 46, 47]
*d*_*PM*_	internalization rate of MCP-1 by macrophages	2.08 × 10^−4^ day^−1^ [40, 46, 47]
*d*_*QQ*_*r*__	binding rate of MMP to TIMP	4.98 × 10^8^ *cm*^3^ *g*^−1^ day^−1^ [40, 46, 47]
*d*_*Q*_*r*_*Q*_	binding rate of TIMP to MMP	1.04 × 10^9^ *cm*^3^ *g*^−1^ day^−1^ [40, 46, 47]
*d*_*Q*_	degradation rate of MMP	4.32 day^−1^ [40, 46, 47]
*d*_*Q*_*r*__	degradation rate of TIMP	21.6 day^−1^ [40, 46, 47]
*d*_*ρQ*_	degradation rate of ECM due to MMP	2.59 × 10^7^ *cm*^3^ *g*^−1^ day^−1^ [40, 46, 47]
*d*_*I*_*γ*__	degradation rate of IFN-*γ*	0.69 day^−1^ [46]
*d*_*S*_	death rate of SMC	0.86 day^−1^ [40, 46, 47]
*d*_*SM*_	apoptosis rate of SMC by macrophages	1.72 day^−1^ fitted
*d*_*I*_12__	degradation rate of IL-12	1.188 day^−1^ [40, 46, 47]
*d*_*T*_*α*__	degradation rate of TNF-*α*	55.45 day^−1^ [40, 46, 47]
*d*_*I*_6__	degradation rate of IL-6	0.173 day^−1^ [43]
*d*_*I*_10__	degradation rate of IL-10	16.64 day^−1^ [47]

**Table 3:** Parameters’ description and value.

Parameter	Description	Value
*χ*_*C*_	chemotactic sensitivity parameter by MCP-1	10 *cm*^5^ *g*^−1^ day^−1^ [40, 46, 47]
*K*_*M*_	macrophage saturation	5 × 10^−5^ *gcm*^−3^ [40]
*K*_*P*_	MCP-1 saturation for influx of macrophages	5 × 10^−9^ *gcm*^−3^ [40]
*K*_*I*_*γ*__	IFN-*γ* saturation for activation of macrophages	1 × 10^−11^ *gcm*^−3^ [40]
*K*_*I*_10__	IL-10 saturation	2 × 10^−7^ g/*cm*^3^ [47]
*K*_*T*_*α*__	TNF-*α* saturation	5 × 10^−7^ g/*cm*^3^ [47]
*ρ*_0_	ECM saturation	10^−3^ *gcm*^−3^ [40]
*M*_0_	source/influx of macrophages from blood	5 × 10^−5^ *gcm*^−3^ [40]
*T*_0_	source/influx of T cells into intima	1 × 10^−3^ *gcm*^−3^ [40]
*P*_0_	MCP-1 concentration	3 × 10^−10^ [40]
$\tilde{\alpha }$	influx rate of macrophages into interstitium	0.2 *cm*^−1^ [40]
*α*_*T*_	influx rate of T cell into interstitium	0.2 *cm*^−1^ [40]
*α*_*I*_6__	influx rate of IL-6 into interstitium	0.2 *cm*^−1^ [40]
*α*_*P*_	influx rate of MCP-1 into interstitium	0.2 *cm*^−1^ [40]
*γ*	influx rate of media into adventitia	0.1 *cm*^−1^ [40]
*S*_0_	initial steady state of SMCs	6 × 10^−3^ *gcm*^−3^ [40]
*I*_60_	source/influx of IL-6	6 × 10^−9^ ∼ 6 × 10^−8^ *gcm*^−3^ [39]
*β*_10_	Coefficient of AAA tissue material properties	17.4 *N*/*cm*^2^ [42] and estimated
*β*_20_	Coefficient of AAA tissue material properties	188.1 *N*/*cm*^2^ [42] and estimated
*k*_1_	Coefficient of AAA tissue material properties	30.4 *N*/*cm*^2^ [42] and estimated
*k*_2_	Coefficient of AAA tissue material properties	84 *N*/*cm*^2^ [42] and estimated
*β*_*p*_	pressure parameter	18.0 *N*/*cm*^2^ [44] and estimated

### *λ*_*I*_6_*M*_ and *λ*_*I*_6_*S*_

According to [16], 10^6^ macrophages produced 10 ng/ml of IL-6. Assuming steady state, we have

$$\begin{array}{c} \lambda_{I_{6}M}M-d_{I_{6}}I_{6}=0. \end{array}$$

Since *d*_*I*_6__ = 0.173 day^−1^ [43], we get *λ*_*I*_6_*M*_ = 1.73 × 10^−6^ day^−1^.

According to [18], 4 × 10^4^ cells/ml SMCs produced 4 ng/ml of IL-6. Assuming steady state, we have

$$\begin{array}{c} \lambda_{I_{6}S}S-d_{I_{6}}I_{6}=0. \end{array}$$

Since *d*_*I*_6__ = 0.173 day^−1^, we get *λ*_*I*_6_*S*_ = 1.73 × 10^−5^ day^−1^.

### *β*_10_, *β*_20_, *k*_1_ and *k*_2_

By [42], *β*_1_ = 0.022 *N*/*mm*^2^ and *β*_10_ = 0.174 *N*/*mm*^2^. We assume that in aneurysm S is approximately half its normal density, i.e., $S\approx \frac{S_{0}}{2}$. Then by Eq (17),

$$\begin{array}{c} \beta_{1}=\beta_{10}-k_{1}\frac{1}{2}, \end{array}$$

which gives *k*_1_ = 30.4 *N*/*cm*^2^.

Similarly, by [42], *β*_2_ = 1.461 *N*/*mm*^2^ and *β*_20_ = 1.881 *N*/*mm*^2^ so that

$$\begin{array}{c} \beta_{20}-k_{2}\frac{1}{2}=\beta_{2}=1.461\;N/mm^{2}; \end{array}$$

hence *k*_2_ = 84 *N*/*cm*^2^.

### *β*_*p*_

By [44], the peak wall stress measured for normal people and AAA patients were 30-44 *N*/*cm*^2^ and 28-37 *N*/*cm*^2^, respectively. We assume that ECM in AAA patients is reduced to 75% of its value in healthy individuals. Hence

$$\begin{array}{c} p^{*}=\frac{30+44}{2}=37\;N/cm^{2},\;\text{and}\;p\left(\frac{3\rho^{*}}{4}\right)=\frac{28+37}{2}, \end{array}$$

which implies, by Eq (18), that *β*_*p*_ = 18 *N*/*cm*^2^.

### 1.1 *σ*_*B*_

We assume *σ*_*B*_ = −*p*_*B*_ *I*, where *p*_*B*_ is average central aortic systolic pressure (100-120 mm Hg) [45]; we take *p*_*B*_ = 110 mm Hg.

## Numerical methods

### Weak formulation

In this subsection, we rewrite in compact form the coupled biological and mechanical systems. We denote by *Z* the vector of the densities or concentrations of cells and cytokines, i.e. *Z* = (*P*, *M*, *T*, *S*, *I*_6_, *I*_10_, *I*_12_, *T*_*α*_, *I*_*γ*_, *Q*, *Q*_*r*_, *ρ*)^*T*^. The entries of *Z* satisfy advection-diffusion equations with nonlinear source term, as seen from Eqs (1)–(12):

$$\begin{array}{c} D_{t}Z+\left(\nabla \cdot v\right)Z-\nabla \cdot \left(D_{Z}\nabla Z\right)=F\left(Z\right),\;\;\text{in}\;\Omega_{M}\left(t\right)\cup \Omega_{A}\left(t\right), \end{array}$$ (22)

where the velocity **v** is defined by **v** = *D*_*t*_ **u**, where *D*_*t*_ denotes the material derivative,

$$\begin{array}{c} D_{t}=\partial_{t}+v\cdot \nabla . \end{array}$$

In the equation for *Z*, *F*(*Z*) is a source term and *D*_*Z*_ is a diagonal matrix with the diagonal terms for the diffusion coefficients of the biological species.

Some of the components of *Z* live only in either Ω_*M*_(*t*) or Ω_*A*_(*t*). Since there is a jump for *Z* at the media/adventitia interface, Γ_*M*_(*t*), we split *Z* into two parts

$$\begin{array}{c} Z_{M}={Z|}_{\Omega_{M}\left(t\right)}\;\;\text{and}\;\;Z_{A}={Z|}_{\Omega_{A}\left(t\right)}. \end{array}$$

The boundary conditions can then be written in the form

$$\begin{array}{c} \left\{ \begin{array}{cc} \frac{\partial Z_{A}}{\partial n}=0, & \;\text{on}\;\Gamma_{A}\left(t\right),\\ \frac{\partial Z_{M}}{\partial n}=\tilde{\gamma }\left(Z_{0}-Z_{M}\right), & \;\text{on}\;\Gamma_{B}\left(t\right),\\ \frac{\partial Z_{M}}{\partial n}=\tilde{\gamma }\left(Z_{A}-Z_{M}\right), & \;\text{on}\;\Gamma_{M}\left(t\right),\\ \frac{\partial Z_{A}}{\partial n}=\tilde{\gamma }\left(Z_{M}-Z_{A}\right), & \;\text{on}\;\Gamma_{M}\left(t\right) \end{array}\right. \end{array}$$

for an appropriate coefficient $\tilde{\gamma }$, some of its component being zero.

The governing equation for the displacement **u** can be written as

$$\begin{array}{ccc} \nabla \cdot \sigma (u) & =0, & \;\text{in}\;\Omega_{M}\left(t\right)\cup \Omega_{A}\left(t\right), \end{array}$$ (23)

where the stress *σ* is is defined in terms of the displacement by

$$\begin{array}{c} \sigma \left(u\right)=-p\left(\rho \right)I+(2\beta_{1}\left(S\right)+4\beta_{2}\left(S\right)\left(\text{tr}\left(B\right)-3\right))B. \end{array}$$

with the boundary conditions

$$\begin{array}{c} \left\{ \begin{array}{ccc} u & =0, & \;\text{on}\;\Gamma_{0},\\ u_{M} & =u_{A}, & \;\text{on}\;\Gamma_{M}\left(t\right),\\ \sigma n & =0, & \;\text{on}\;\Gamma_{0}\cup \Gamma_{A}\left(t\right),\\ \sigma n & =\sigma_{B}n-\gamma \kappa n, & \;\text{on}\;\Gamma_{B}\left(t\right),\\ {\left(\sigma n)\right|}_{\Omega_{M}} & ={\left(\sigma n)\right|}_{\Omega_{A}}+\gamma \kappa n & \;\text{on}\;\Gamma_{M}\left(t\right). \end{array}\right. \end{array}$$

Multiplying the coupled System (22) and (23) by arbitrary test functions (*W*_*M*_, *W*_*A*_,**w**), and performing integration by parts using the boundary conditions, we get the weak formulation:

$$\begin{array}{c} \left\{ \begin{array}{ccc} & {\left(D_{t}Z_{M},W_{M}\right)}_{\Omega_{M}\left(t\right)}+{\left(\left(\nabla \cdot v\right)Z_{M},W_{M}\right)}_{\Omega_{M}\left(t\right)}+{\left(D_{Z}\nabla Z_{M},\nabla W_{M}\right)}_{\Omega_{M}\left(t\right)} & \\ & -{\left\langle \gamma \left(Z_{A}-Z_{M}\right),W_{M}\right\rangle }_{\Gamma_{M}\left(t\right)}-{\left\langle \alpha_{Z}\left(Z_{0}-Z_{M}\right),W_{M}\right\rangle }_{\Gamma_{B}\left(t\right)}={\left(F,W_{M}\right)}_{\Omega_{M}\left(t\right)}, & \\ & {\left(D_{t}Z_{A},W_{A}\right)}_{\Omega_{A}\left(t\right)}+{\left(\left(\nabla \cdot v\right)Z_{A},W_{A}\right)}_{\Omega_{A}\left(t\right)}+{\left(D_{Z}\nabla Z_{A},\nabla W_{A}\right)}_{\Omega_{A}\left(t\right)} & \\ & -{\left\langle \gamma \left(Z_{M}-Z_{A}\right),W_{A}\right\rangle }_{\Gamma_{M}\left(t\right)}={\left(F,W_{A}\right)}_{\Omega_{A}\left(t\right)}, & \\ & {\left(\sigma ,\nabla w\right)}_{\Omega (t)}-{\left\langle \sigma_{B}n-\gamma \kappa n,w\right\rangle }_{\Gamma_{B}\left(t\right)}+{\left\langle \gamma \kappa n,w\right\rangle }_{\Gamma_{M}\left(t\right)}=0. & \end{array}\right. \end{array}$$ (24)

Here we used (⋅, ⋅)_*D*_ and 〈⋅, ⋅〉_∂*D*_ to denote the *L*^2^ inner product on the domain *D* and on the boundary ∂*D*, respectively.

### Lagrangian description for hyperelastic equation

Ω_*A*_(*t*), Ω_*M*_(*t*) and their boundaries are moving in time. We set Ω(*t*) = Ω_*M*_(*t*) ∪ Ω_*A*_(*t*) ∪ Γ_*M*_(*t*) and ${\hat{\Omega }}_{M}=\Omega_{M}\left(0\right)$, ${\hat{\Omega }}_{A}=\Omega_{A}\left(0\right)$, $\hat{\Omega }=\Omega \left(0\right)$. To characterize the motion of the media and adventitia, we introduce a flow map $x(\hat{x},t)$ for the position of the particle $\hat{x}$ at time *t*.

Then each function can be described in Lagrangian coordinates, namely for each function *f*(*x*, *t*), its Lagrangian description is

$$\begin{array}{c} \hat{f}\left(\hat{x},t\right)=f\left(x\left(\hat{x},t\right),t\right). \end{array}$$

We also introduce the following variables in Lagrangian coordinates: The displacement $\hat{u}\left(\hat{x},t\right)=u\left(x\left(\hat{x},t\right),t\right)=x\left(\hat{x},t\right)-\hat{x}$, the deformation tensor $F\left(\hat{x},t\right)=\frac{\partial x}{\partial \hat{x}}\left(\hat{x},t\right)=I+\frac{\partial \hat{u}}{\partial \hat{x}}\left(\hat{x},t\right)$, and its determinant $J\left(\hat{x},t\right)=\text{det}\left(F\left(\hat{x},t\right)\right)$.

By change of variables, we can write the weak form for the equilibrium equation for **w** in Lagrangian coordinates,

$$\begin{array}{c} {\left(J\hat{\sigma }F^{-T},\hat{\nabla }\hat{w}\right)}_{\hat{\Omega }}-{\left\langle J{\hat{\sigma }}_{B}F^{-T}\hat{n},\hat{w}\right\rangle }_{\Gamma_{B}\left(0\right)}={\left\langle \gamma \kappa n,w\right\rangle }_{\Gamma_{M}\left(t\right)\cup \Gamma_{B}\left(t\right)} \end{array}$$ (25)

where

$$\begin{array}{c} \hat{\sigma }=-J\hat{p}F^{-T}+J(\beta_{1}\left(\hat{S}\right)+2\beta_{2}\left(\hat{S}\right)\left(\text{tr}\left(B\right)-3\right))F. \end{array}$$

As for the surface tension term, we can also write it in the Lagrangian coordinates as follows:

$$\begin{array}{c} {\left\langle \gamma \kappa n,w\right\rangle }_{\Gamma (t)}=\int_{\hat{\Gamma }}\gamma J\left|F^{-T}\hat{n}\right|(\text{tr}\left(\hat{\nabla }wF^{-1}\right)-\frac{{\hat{n}}^{T}F^{-1}\hat{\nabla }wF^{-1}F^{-T}\hat{n}}{{|F^{-T}\hat{n}|}^{2}})\;d\hat{S}. \end{array}$$ (26)

By substituting this expression into the hyperelasitcity equation in Lagrangian coordinates Eq (25), we can get a nonlinear equation:

$$\begin{array}{c} a_{L}\left(\hat{u},\hat{w};\hat{S},\hat{\rho }\right)=0 \end{array}$$ (27)

### Finite element discretization on the moving mesh

In order to deal with the free boundary and the jump at the interface, we use the moving mesh method.

The initial domains ${\hat{\Omega }}_{M}$ and ${\hat{\Omega }}_{A}$ are discretized by triangulations, denoted by $T_{M,h}^{0}$ and $T_{A,h}^{0}$, respectively. We consider the case that $T_{M,h}^{0}$ and $T_{A,h}^{0}$ are matching on the interface $\Gamma_{M}^{0}$. We use piecewise linear and continuous function ${\hat{u}}_{h}$ to approximate the displacement. Then the triangulation $T_{M,h}^{n}\cup T_{A,h}^{n}$ for the current domain $\Omega_{h}^{n}$ can be obtained by moving the grid nodes of $T_{M,h}^{0}\cup T_{A,h}^{0}$ according to the flow map $x\left(\hat{x},t\right)=\hat{x}+{\hat{u}}_{h}\left(\hat{x},t\right)$.

We next introduce the following time discretization for the material derivative

$$\begin{array}{c} {\left(\hat{D_{t}Z}\right)}^{n}\approx {\left(\hat{D_{t,k}Z}\right)}^{n}:=\frac{Z\left(x\left(\hat{x},t^{n}\right),t^{n}\right)-Z\left(x\left(\hat{x},t^{n-1}\right),t^{n-1}\right)}{k}, \end{array}$$

and for

$$\begin{array}{c} {\hat{v}}^{n}={\hat{D_{t}u}}^{n}\approx \frac{\hat{u}\left(\hat{x},t^{n}\right)-\hat{u}\left(\hat{x},t^{n-1}\right)}{k} \end{array}$$

where we use the superscript *n* to indicate the function at time *nk*, where *k* is the time step. Note that no interpolation is needed for evaluating the material derivative *D*_*t*,*k*_ at grid points, since the grid points are moving according to the flow map.

For the space discretization, we use piecewise linear and continuous function spaces $V_{h},W_{h,M}^{n}$ and $W_{h,A}^{n}$ to approximate the solutions. Let ${\left\{{\hat{w}}_{i}\right\}}_{i=1}^{N}$, ${\left\{W_{M,i}^{n}\right\}}_{i=1}^{N_{M}}$ and ${\left\{W_{A,i}^{n}\right\}}_{i=1}^{N_{A}}$ be the bases of $V_{h},W_{h,M}^{n}$ and $W_{h,A}^{n}$, respectively, where *N*, *N*_*M*_ and *N*_*A*_ are the numbers of basis functions of the corresponding spaces. The approximation solutions at time *nk* can be written as summations

$$\begin{array}{c} Z_{M,h}^{n}=\sum_{i=1}^{N_{M}}c_{M,i}^{n}W_{M,i}^{n}, \end{array}$$

$$\begin{array}{c} Z_{A,h}^{n}=\sum_{i=1}^{N_{A}}c_{A,i}^{n}W_{A,i}^{n}, \end{array}$$

$$\begin{array}{c} {\hat{u}}_{h}^{n}=\sum_{i=1}^{N}c_{i}^{n}{\hat{w}}_{i}^{n}, \end{array}$$

with coefficients to be determined by the discrete system, corresponding to the System (24),

$$\begin{array}{c} \left\{ \begin{array}{ccc} & {\left({\left(D_{t,k}Z_{M}\right)}_{h}^{n},W_{M}\right)}_{\Omega_{M}^{n}}+{\left(\left(\nabla \cdot v_{h}^{n}\right)Z_{M,h}^{n},W_{M}\right)}_{\Omega_{M}^{n}}+{\left(D_{Z}\nabla Z_{M,h}^{n},\nabla W_{M}\right)}_{\Omega_{M}^{n}} & \\ & -{\left\langle \gamma \left(Z_{A,h}^{n}-Z_{M,h}^{n}\right),W_{M}\right\rangle }_{\Gamma_{M}^{n}}-{\left\langle \alpha_{Z}\left(Z_{0}-Z_{M,h}^{n}\right),W_{M}\right\rangle }_{\Gamma_{B}^{n}}={\left(F_{h}^{n-1},W_{M}\right)}_{\Omega_{M}^{n}}, & \\ & {\left({\left(D_{t,k}Z_{A}\right)}_{h}^{n},W_{A}\right)}_{\Omega_{A}^{n}}+{\left(\left(\nabla \cdot v_{h}^{n}\right)Z_{A,h}^{n},W_{A}\right)}_{\Omega_{A}^{n}}+{\left(D_{Z}\nabla Z_{A,h}^{n},\nabla W_{A}\right)}_{\Omega_{A}^{n}} & \\ & -{\left\langle \gamma \left(Z_{M,h}^{n}-Z_{A,h}^{n}\right),W_{A}\right\rangle }_{\Gamma_{M}^{n}}={\left(F_{h}^{n-1},W_{A}\right)}_{\Omega_{A}^{n}}, & \\ & a_{L}\left({\hat{u}}_{h}^{n},\hat{w};{\hat{S}}_{h}^{n-1},{\hat{\rho }}_{h}^{n-1}\right)=0, \end{array}\right. \end{array}$$ (28)

for any $\hat{w}\in V_{h},W_{M}\in W_{h,M}^{n}$ and $W_{A}\in W_{h,A}^{n}$. Here we use the explicit discretization of the coefficients for the elasticity, and the source terms for the biological species. We denote the first two equations in Eulerian coordinates of the System (28) by

$$\begin{array}{c} a_{E}\left(Z_{h}^{n},W;Z_{h}^{h-1},v_{h}^{n},F_{h}^{n-1},\Omega_{h}^{n}\right)=0. \end{array}$$

By this discretization, the elastic equation and the biological equation are decoupled. Given the solutions in last time step, we use the Newton’s nonlinear iteration method to solve the system *a*_*L*_ = 0 in the initial domain. Then we update the triangulations for the current domain $\Omega_{h}^{n}$ by the approximate flow map $x\left(\hat{x},t\right)=\hat{x}+{\hat{u}}_{h}\left(\hat{x},t\right)$. Following the standard technique of finite element method, we then solve the variational problem *a*_*E*_ = 0 for the biological species. We thus get the solution of current time step.
